# A new purification technique to obtain specific size distribution of giant lipid vesicles using dual filtration

**DOI:** 10.1371/journal.pone.0254930

**Published:** 2021-07-29

**Authors:** Mohammad Abu Sayem Karal, Tawfika Nasrin, Marzuk Ahmed, Md. Kabir Ahamed, Shareef Ahammed, Salma Akter, Sharif Hasan, Zaid Bin Mahbub

**Affiliations:** 1 Department of Physics, Bangladesh University of Engineering and Technology, Dhaka, Bangladesh; 2 Department of Mathematics and Physics, North South University, Dhaka, Bangladesh; King Abdulaziz University, SAUDI ARABIA

## Abstract

A new purification technique is developed for obtaining distribution of giant unilamellar vesicles (GUVs) within a specific range of sizes using dual filtration. The GUVs were prepared using well known natural swelling method. For filtration, different combinations of polycarbonate membranes were implemented in filter holders. In our experiment, the combinations of membranes were selected with corresponding pore sizes–(i) 12 and 10 μm, (ii) 12 and 8 μm, and (iii) 10 and 8 μm. By these filtration arrangements, obtained GUVs size distribution were in the ranges of 6−26 μm, 5–38 μm and 5–30 μm, respectively. In comparison, the size distribution range was much higher for single filtration technique, for example, 6−59 μm GUVs found for a membrane with 12 μm pores. Using this technique, the water-soluble fluorescent probe, calcein, can be removed from the suspension of GUVs successfully. The size distributions were analyzed with lognormal distribution. The skewness became smaller (narrow size distribution) when a dual filtration was used instead of single filtration. The mode of the size distribution obtained in dual filtration was also smaller to that of single filtration. By continuing this process of purification for a second time, the GUVs size distribution became even narrower. After using an extra filtration with dual filtration, two different size distributions of GUVs were obtained at a time. This experimental observation suggests that different size specific distributions of GUVs can be obtained easily, even if GUVs are prepared by different other methods.

## 1. Introduction

Vesicles are model of cells, which are used for studying the function of complex biomembranes [1]. These vesicles are considered as a promising tool for their wide range of industrial and medical applications [2–4]. The cell size vesicles such as giant unilamellar vesicles (GUVs) have been widely used to investigate the elasticity of membranes [5], deformation [6, 7] and poration [8, 9] of vesicles. Although intravenous drug delivery generally uses vesicles with sizes smaller than 1 μm, some clinical applications such as inhaled liposomal drug delivery uses larger vesicles of sizes several microns [10–12]. One of the main advantages of using the GUVs over nanometer-size vesicles is its visibility in optical microscopy, for example, it is possible to observe the static and dynamic changes of GUVs induced by peptides, toxins and nanoparticles [13, 14].

Among the various methods for preparing different sizes GUVs [15], the natural swelling [16] and electroformation [17] method are highly popular for synthesizing oil-free GUVs. During synthesis, many small vesicles, some lipid aggregates and a small fraction of multilamellar vesicles (MLVs) are generally formed in the suspension of GUVs, which is hindering the interaction between membrane active agents and lipid vesicles. Such lipid aggregates and MLVs are removed by the high speed centrifugation of GUV suspension during preparation and left smaller vesicles [16, 18–20]. Therefore, it is necessary to purify the GUVs. The dialysis, a time-consuming technique [21], requires a large volume of external solution to flow the buffer for several hours, whereas centrifugation takes only 10–15 minutes [22], however it requires a high concentration of sucrose and glucose in the inside and outside of GUVs. To obtain oil-free GUVs, membrane filtering with micrometer pores polycarbonate membrane [19] and microfiltration [23] methods are generally used. Those methods can purify the GUVs by eliminating the non-entrapped solute such as nanometer sized vesicles from the suspension. Recently, we have reported a gravity-based non-electromechanical membrane filtering technique [24], which shows similar performance like membrane filtering method. These methods/techniques provide a wide range size distribution of purified GUVs. As an example, if polycarbonate membrane with 10 μm pores is used for purification, the obtained size distribution of GUVs would be 8–70 μm [19, 23, 24]. To our knowledge, so far, all the reported purification techniques produce wide range size distribution GUVs rather than narrow size distribution.

**Fig 1:**
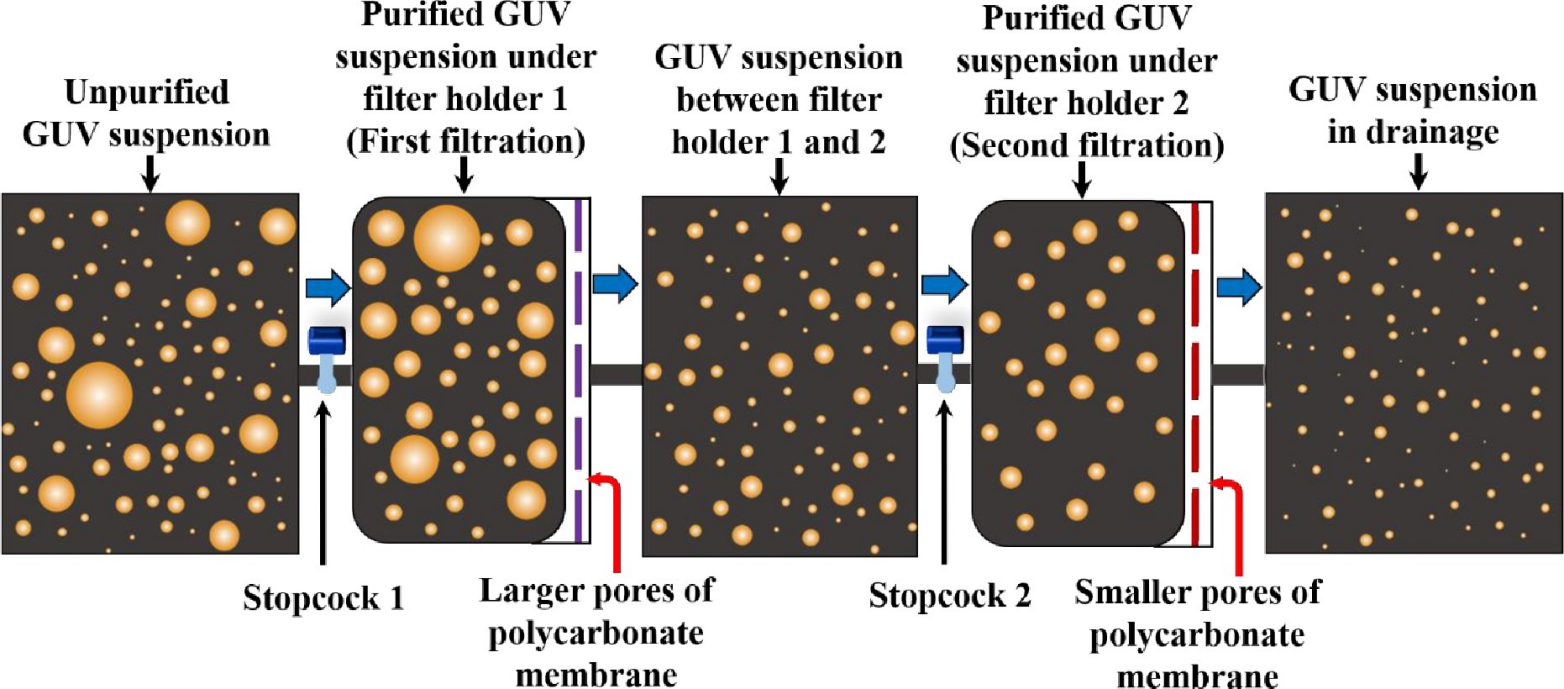
Schematic diagram of dual filtration.

The extrusion-dialysis method [25] was developed for preparing the monodisperse vesicles. The overall duration of the process required ~24 h. Such a longer time period would be hampered the stability of spherical shaped vesicles. It is very important to mention that, in many experiments specific size GUVs are necessary. For example, the ‘single GUV method’ [13, 14] requires similar sizes GUVs, where a ‘single GUV’ is induced by various type of membrane active agents and calculate the corresponding kinetic constant of pore formation and membrane permeation [5, 20]. Moreover, similar size GUVs are also essential for the experiment of peptide-induced pore sizes determination in the membranes [26]. Recently, we investigated the electric tension-induced pore formation in membranes using similar-size GUVs for obtaining the kinetics of poration [27]. Therefore, a specific size distribution of purified GUVs is indispensable for various experiments. To fulfil the target, we developed a new purification technique with dual filtration for getting specific size range of vesicles. Fig 1 shows the schematic diagram on what happens with the suspension of giant vesicles upon the first and the second filtration.

In this novel idea, instead of using a single polycarbonate membrane, a combination of such membranes with different size pores is used. The reason we used the dual filtration technique is to get a specific size distribution of vesicles rather than a wide range size distribution. In dual filtration, three combinations of polycarbonate membranes were implemented in filter holder 1 and filter holder 2. For example: case (i) polycarbonate membrane with 12 μm pores in filter holder 1 and polycarbonate membrane with 10 μm pores in filter holder 2. For case (ii) the corresponding polycarbonate membranes with pores were 12 μm and 8 μm, and for case (iii) the polycarbonate membranes with pores were 10 μm and 8 μm. In each case, polycarbonate membrane with higher pores was implemented in filter holder 1, and smaller pores in filter holder 2 as illustrated in Fig 1. Each arrangement provided a specific size distribution of vesicles. The purified GUV suspension was collected under filter holder 2 as shown in Fig 1. In contrast, in single filtration, only one polycarbonate membrane with pores 12 μm or 10 μm or 8 μm was implemented in filter holder 1. It is to be noted that the successive part such as filter holder 2 was absent in the single filtration. The purified GUV suspension was collected under filter holder 1. Hence, the technique provided a wide range of size distribution of vesicles as depicted in Fig 1. The experimental results are presented in a set of size distribution histograms, and the distributions are analyzed by lognormal distribution. We calculated the average size of GUVs, the skewness and mode of distribution at various conditions.

## 2. Materials and methods

### 2.1 Synthesis of lipid membranes of GUVs

The unilamellar lipid vesicles such as GUVs were prepared using the well-known natural swelling method that was developed in 1969 [16]. From then many researchers in many laboratories have been using this simple but effective method for preparing unilamellar vesicles [5, 24, 28–34]. For synthesis of lipid membranes of GUVs, 1,2-dioleoyl-*sn*-glycero-3-phospho-(1′-*rac*-glycerol) (sodium salt) (DOPG) and 1,2-dioleoyl-*sn*-glycero-3-phosphochorine (DOPC) were purchased from Avanti Polar Lipids Inc. (Alabaster, AL). Bovine serum albumin (BSA), 1,4-Piperazinediethanesulfonic acid (PIPES), Ethylene glycol-bis(2-aminoethylether)-*N*,*N*,*N*′,*N*′-tetraacetic acid (EGTA) and calcein were purchased from Sigma-Aldrich (Germany). 40%DOPG/60%DOPC-GUVs (% indicates mole %) were synthesized by the natural swelling method [16]. At first, 1 mM DOPG and DOPC (total 200 μL) in chloroform were taken into a 4.5 mL glass vial and dried with a gentle flow of nitrogen gas to produce a thin and homogeneous lipid film. The residual chloroform in the film was removed by placing the vial in a vacuum desiccator overnight (12 hours). An amount of 20 μL MilliQ water was added into the vial and then pre-hydrated at 45°C for 8 minutes. After pre-hydration, the sample was incubated with 1 mL of buffer A (10 mM PIPES, pH 7.0, 150 mM NaCl, 1 mM EGTA containing 0.10 M sucrose) for 3 hours at 37°C to produce unpurified GUV suspension. For preparing the water-soluble fluorescent probe, calcein, containing GUVs, vesicles were synthesized in the buffer A containing 1 mM calcein in the inside of vesicles.

### 2.2 Method of purifying the GUVs

**Fig 2:**
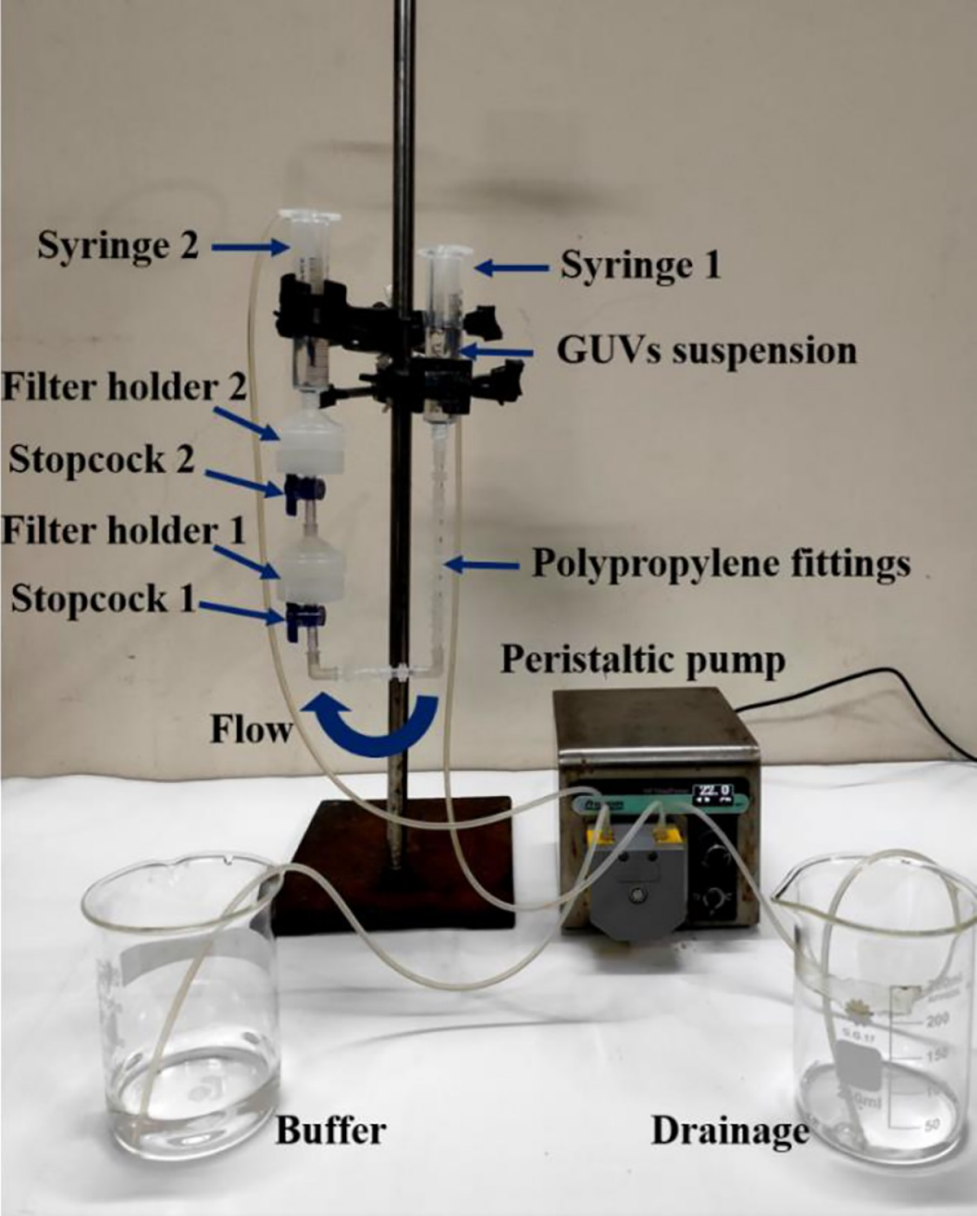
Experimental set-up of dual filtration technique.

As a new technique of purification, we used combinations of polycarbonate membranes with different pore sizes as shown in Fig 2. The arrangement and steps we followed are discussed below. The incubated unpurified GUV suspension was centrifuged at 13,000×g (here g is the acceleration due to gravity) for 20 minutes at 20°C using a refrigerated centrifuge (NF 800R, NUVE, Turkey). The function of the centrifugation is to remove the multilamellar vesicles (MLVs) and lipid aggregates as these elements sedimented at the bottom of eppendorf tubes [18–20]. After centrifugation, an amount of 2.6−2.8 mL supernatant (unpurified GUV suspension) was collected for purification experiment. Therefore, most of the lipid aggregates and MLVs left at the bottom of eppendorf tubes as we collected the supernatant after centrifugation. Then the unpurified GUV suspension in buffer B (10 mM PIPES, pH 7.0, 150 mM NaCl, mM EGTA containing 0.10 M glucose) was added to a 10 mL syringe 1 (JMI Syringes and Medical Devices Ltd. Bangladesh) where the buffer B was continuously flowing by a peristaltic pump (CPP-SP2, Shenchen, China) through a plastic tube of inner diameter 3 mm. The syringe 1 was connected to bottom of filter holder 1 (Swinnex, *ϕ* = 25 mm, Millipore Co., Billerica, MA) through a tube prepared with three different types of polypropylene fittings (Luer fittings VRFE6, VRFC6, VRSC6; AS-ONE, Japan) having inner diameter 3 mm. The tube contains a total number of 18 fittings. The upper end of filter holder 1 was connected to bottom of filter holder 2, and the upper end of filter holder 2 was connected to a 10 mL plastic syringe 2 (of same company). The polycarbonate membranes (Whatman® Nuclepore™ Track-Etched Membranes, UK) were set inside the filter holders. The arrangement of that membranes was followed in descending order of pore sizes in filter holders 1 and 2, respectively. We arranged three combinations of polycarbonate membranes in filter holder 1 and filter holder 2 according to corresponding pore sizes such as case (i) holder 1: 12 μm and holder 2: 10 μm, case (ii) holder 1: 12 μm and holder 2: 8 μm, and case (iii) holder 1: 10 μm and holder 2: 8 μm. One important thing is that prior to flow of buffer B into syringe 1, it is necessary to remove air bubbles from the polypropylene tube and the filter holders. If the bubbles were present, it hindered the purification process.

For that reason, before starting the experiment, we poured 5−6 mL buffer B into syringe 1. We inserted a pasteur pipette into the tube passing through that syringe 1. Then push and pull the pasteur pipette for several times so that air bubbles came to the pipette and removed. Complete removal was confirmed by checking the similar height of buffer B in the syringe 1 and 2. The flow rate 1 mL/min was controlled by the peristaltic pump. The GUV suspension passed through the pores of polycarbonate membrane to syringe 2. A 1-way polycarbonate stopcock (Luer stopcock VXB1055, AS-ONE, Japan) was inserted between the filter holder 1 and the polypropylene tube, and also between the filter holder 1 and 2 as shown in Fig 2. After filtering the GUV suspension for 1 h, the stopcock was closed along with stopped the peristaltic pump and the solution in the space under filter holder 2 was used as purified GUV suspension for dual filtration. The time duration of about 1 h was necessary to purify the GUV suspension. Otherwise, a large population smaller vesicle of sizes less than 3 μm remained in the purified GUV suspension. In addition, for complete removal of fluorescent probe (calcein) from the GUV suspension, it was also necessary to run the purification process for about 1 h. Although microfiltration [23] required shorter time (~ 10 min) for purification, the recovery rate was relatively low (< 50%).

**Fig 3:**
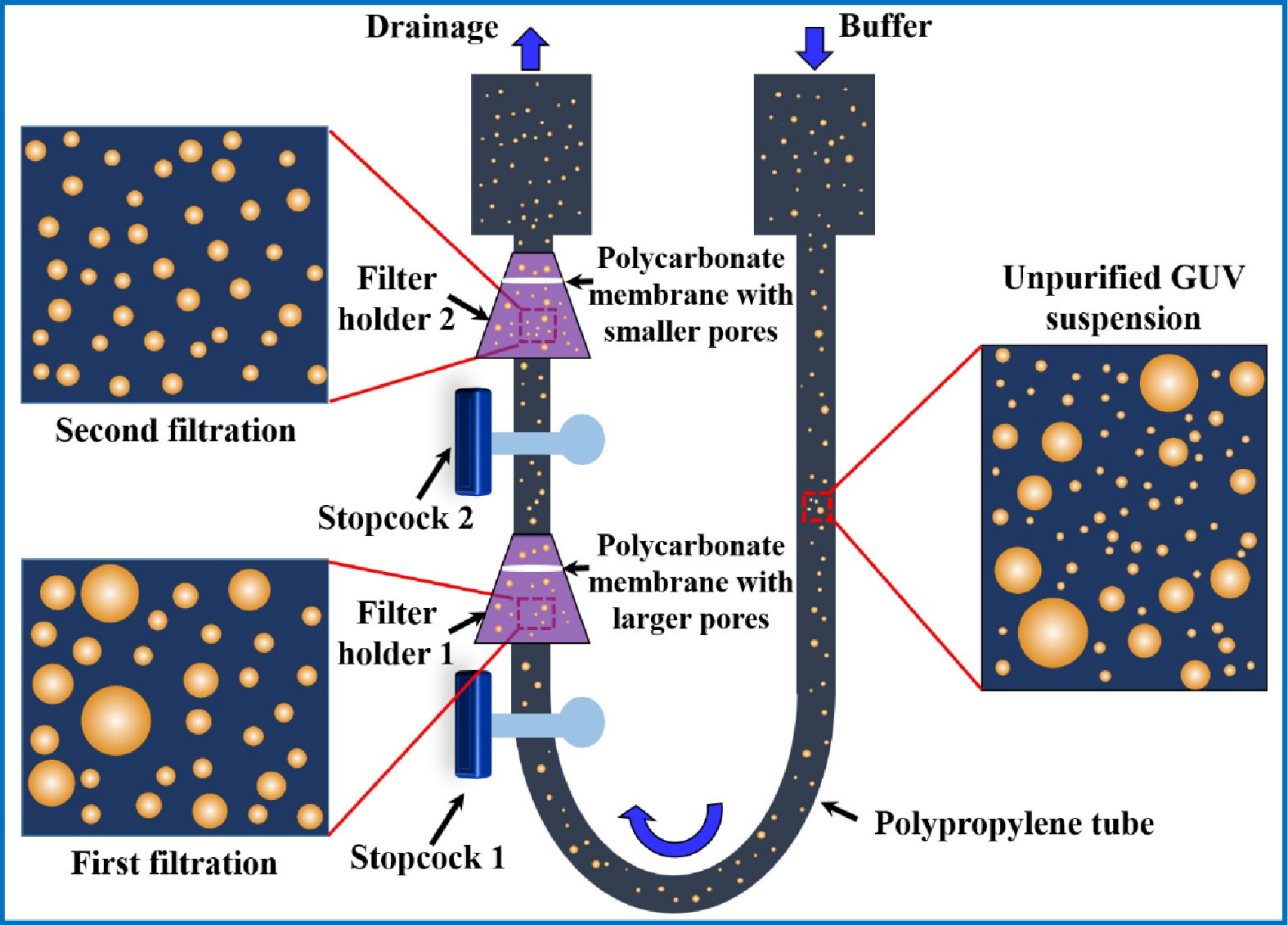
A schematic representation of experimental set-up of dual filtration.

A schematic representation of experimental set-up of dual filtration is provided in Fig 3. The illustration labelled by several graphical lines such as unpurified GUV suspension, GUV suspension in first filtration and GUV suspension in second filtration. The stopcock 1 and 2, filter holder 1 and 2, polycarbonate membrane with larger and smaller pores, and polypropylene tube are also labelled in the illustration.

**Fig 4:**
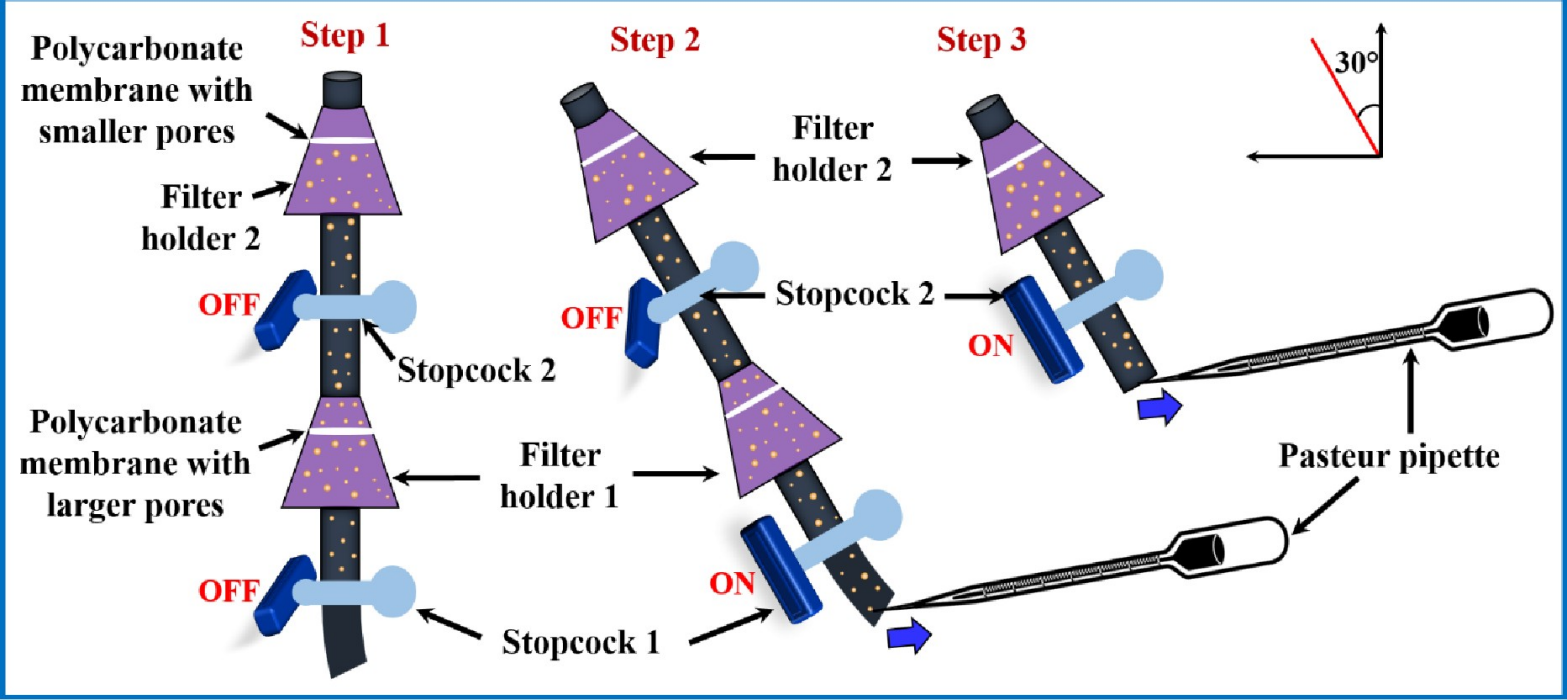
A step wise schematic representation of collecting the purified GUV suspension from the space under filter holder 1 and filter holder 2. Step 2 is the process of collecting the purified GUV suspension for single filtration, while step 3 is the process of collecting the purified GUV suspension for dual filtration.

For the collection of purified GUV suspension from the space under filter holder 1 and filter holder 2, we followed the steps as illustrated in Fig 4. It is to be remembered that the sample collected from filter holder 1 was used as a purified GUV suspension for single filtration, while the sample collected from filter holder 2 was used as a purified GUV suspension for dual filtration. In step 1, both the stopcocks were closed as purification process was completed. We kept the filter holder and stopcock combination at inclined position (30-degree angle) and inserted a pasteur pipette at the bottom of stopcock 1, and collected the purified GUV suspension slowly (by sucking) from the space under filter holder 1 by opening the stopcock 1 (step 2 of Fig 4). In step 3, we removed filter holder 1 with polypropylene tube from the lower end of stopcock 2. Then collected the purified GUV suspension for dual filtration by opening the stopcock 2 and suck the purified solution slowly. We kept the purified solutions into 4 eppendorf tubes for further experiment.

**Fig 5:**
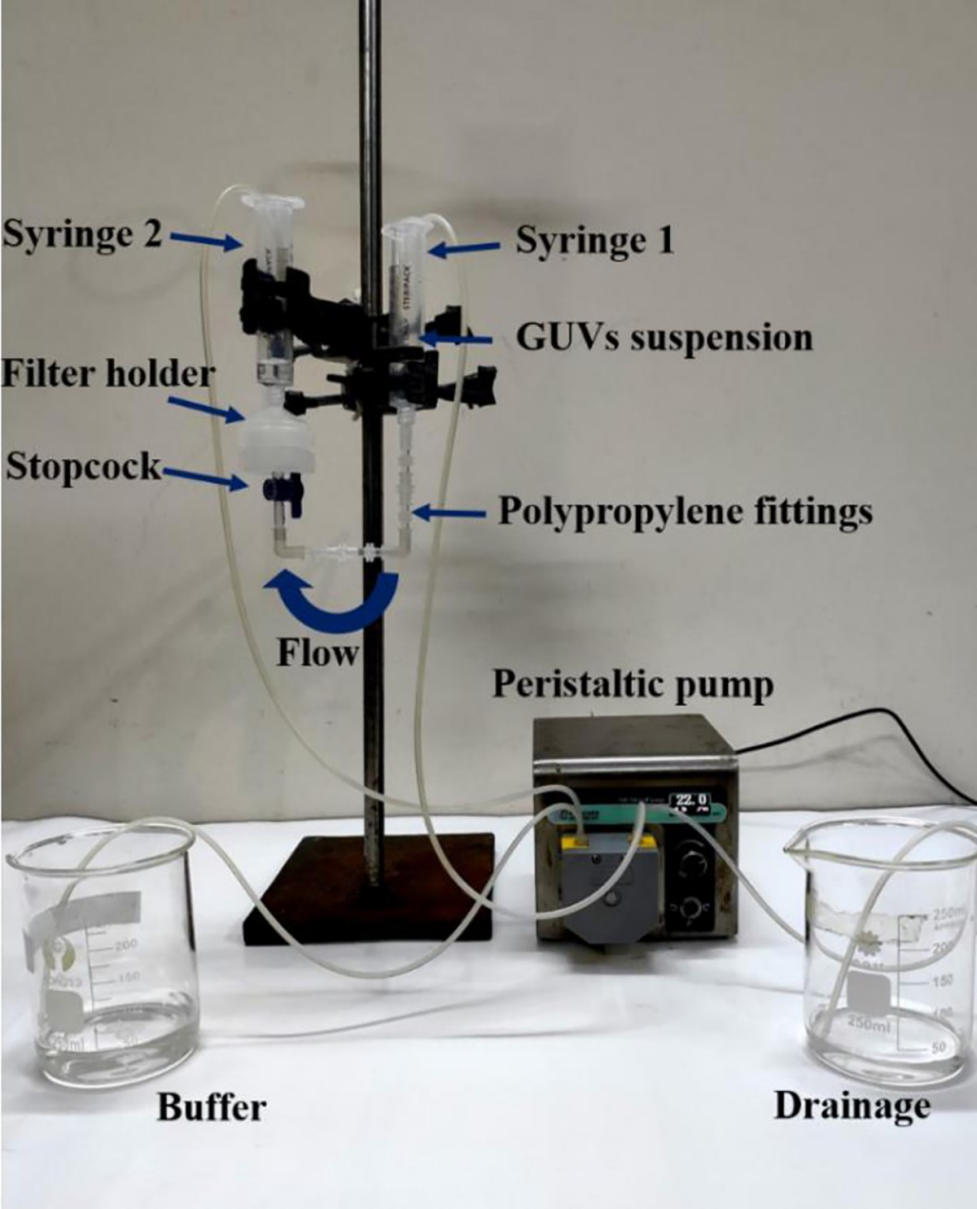
Experimental set-up of single filtration technique.

As a comparison of dual filtration with single filtration, the experimental set-up is provided in Fig 5. In this technique, only one filter holder with polycarbonate membrane was used as shown in Fig 5. In this case, the tube contains a total number of 9 fittings. The polycarbonate membranes were used in the filter holder with pore sizes 12 or 10 or 8 μm. The process of collecting the purified GUV suspension for single filtration is depicted in step 2 of Fig 4. After finishing the purification, an amount of 300 μL GUV suspension was taken into a handmade microchamber for microscopic observation. The inside of microchamber was coated with 0.10% (w/v) BSA dissolved in buffer B to restrict the interaction between the glass surface and GUVs. For observing the purified GUVs in suspension, buffer A used as an internal solution, while buffer B used as an external solution of GUVs. The GUVs were observed and recorded with a charged couple device camera using an inverted phase contrast fluorescence microscope (IX-73, Olympus, Japan) with 20× objective at 25 ± 1°C. All the data after dual filtration [case (i), case (ii) and case (iii)] and single filtration were collected and analyzed by appropriate mathematical tools and sequentially mentioned in sections 3.1−3.3. It is worth to mention that the ability of removing the water-soluble fluorescent probe (calcein) from the suspension of GUVs by the dual filtration technique was observed. The effects of flow rate on the average size of GUVs in dual filtration technique was investigated to obtain the suitable flow rate.

We calculated the fraction of required sizes GUVs under filter holder 2 compared to the total number of GUVs before filtration. In order to assess the efficiency of the process, we observed the effects of repeating the dual filtration on the size distribution of GUVs. In that case, a new set of membranes was used in the filter holders before repeating the experiment. We observed the purification of GUVs using an extra filtration with dual filtration as well. The results of these observations were sequentially presented in sections 3.4−3.8.

## 3. Results

Microscopic observations of purified GUVs from dual and single filtration techniques and their size distribution analysis using lognormal distribution are describing below.

### 3.1. Dual filtration with a combination of 12 and 10 μm pores polycarbonate membranes

**Fig 6:**
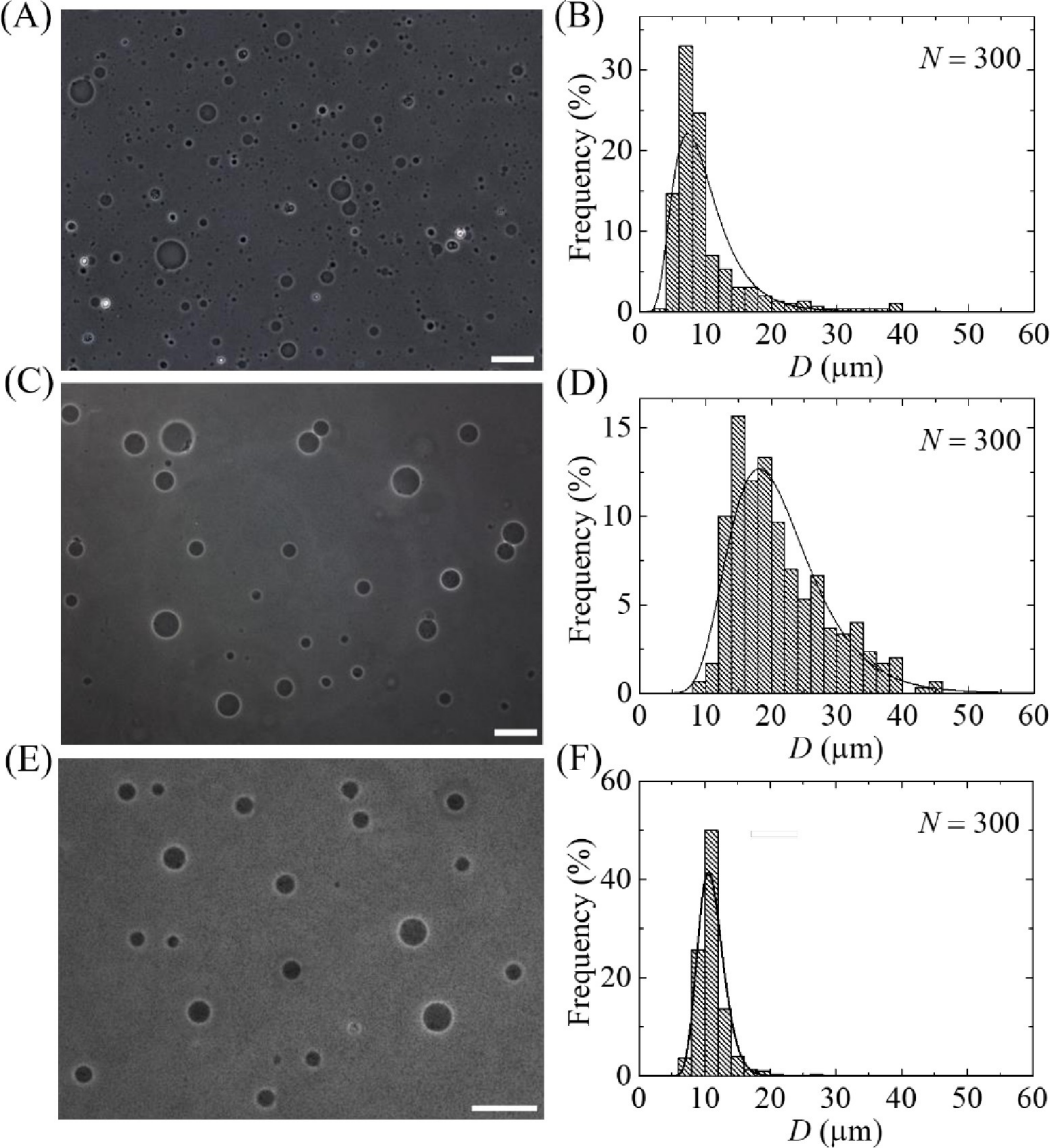
Effects of dual filtration with 12 and 10 μm (case i) pores polycarbonate membranes on the size and distribution of 40%DOPG/60%DOPC-GUVs. (A) and (B) show the phase contrast image and the corresponding size distribution histogram of unpurified GUVs. (C) and (D) show the phase contrast image and the corresponding size distribution histogram of GUVs in filter holder 1. (E) and (F) show the phase contrast image and the corresponding size distribution histogram of GUVs in filter holder 2. The purification was performed at flow rate 1 mL/min for 1 h. The bar in the images (A, C, E) corresponds to a length of 50 μm. The solid line of B, D and F corresponds to fitting with Eq 1.

This section is demonstrating the effects of dual filtration by showing the images, size distributions and corresponding analysis of unpurified and purified GUVs. The unpurified GUV suspension was added into the 60 mL buffer B that was continuously flowing from syringe 1 to filter holder 1 by a peristaltic pump. Hence, the volume ratio of purified GUV suspension and buffer B was 1:22. On the other hand, for getting the image of unpurified GUV suspension, an amount of 20 μL unpurified GUV suspension was diluted with 280 μL buffer B solution in the microchamber, which provided the volume ratio of unpurified GUV suspension and buffer B 1:14. To investigate the effects of dual filtration with a combination of polycarbonate membranes on the size and distribution of GUVs, 12 μm pores polycarbonate membrane was used in filter holder 1 and 10 μm pores polycarbonate membrane was used in filter holder 2 (case i). Fig 6A shows a phase contrast image of unpurified GUV suspension, showing different sizes of GUVs. Fig 6B shows the corresponding histogram of the size (i.e., diameter, *D*) distribution of 300 GUVs (i.e., number of measured GUVs, *N* = 300), where the range of size of GUVs was 3.7−38.7 μm. A similar result was also obtained for other independent experiments (i.e., number of independent experiment, *n* = 3). Fig 6C shows the phase contrast image of GUVs obtained under filter holder 1. Fig 6D shows the corresponding size distribution histogram of same *N*, where the range of size of GUVs was 9.2−45.0 μm. Fig 6E shows the phase contrast image of GUVs obtained under filter holder 2. Fig 6F shows the corresponding size distribution histogram of same *N*, where the range of size of GUVs was 6.6−26.2 μm. These results clearly indicate that a wide range of size distribution was observed when GUVs were collected under filter holder 1 since it was the first step of dual filtration technique. In contrast, the size distribution became narrow under filter holder 2. It means that GUVs were filtered for both 12 μm and 10 μm pores polycarbonate membrane.

We analyzed the size distribution histograms with the well-known lognormal distribution [35], which was also previously used for fitting the size distribution of GUVs [24, 36]. The lognormal distribution is described as follows:

$$f(D)=\frac{1}{D\sigma \sqrt{2\pi }}\exp[-\frac{{\left(\ln D-\mu \right)}^{2}}{2\sigma^{2}}]$$ (1)

where, *f*(*D*) indicates the normalized counts of GUVs with diameter *D*, *μ* is the mean and *σ*^2^ is the variance. Then the average value of the distribution is as follows:

$$d_{\mathrm{ave}}=\exp(\mu +\frac{1}{2}\sigma^{2})$$ (2)

In the lognormal distribution, the skewness measures the asymmetry of the probability distribution about its mean position. The skewness of the distribution is as follows:

$$\chi =[\exp(\sigma^{2})+2]\sqrt{\exp(\sigma^{2})-1}$$ (3)

Generally, mode is the value that is most likely obtained in the samples. In lognormal distribution, it is expressed as follows:

$$X=\exp(\mu -\sigma^{2})$$ (4)

All the histograms of Fig 6 were fitted with Eq 1, and then the average value (*d*_ave_), skewness (*χ*) and mode (*X*) of the distribution were obtained using Eqs 2, 3 and 4, respectively. The corresponding values of coefficient of determination, *R*^2^, were also calculated to ensure the best fittings. In the first independent experiment, the values of *d*_ave1_ were 9.9 μm for unpurified GUVs, 21.4 μm for purified GUVs under filter holder 1 and 11.1 μm for purified GUVs under filter holder 2. The values of corresponding skewness *χ*_1_ were 1.52, 1.05 and 0.55. Similarly, the values of corresponding mode were *X*_1_ were 7.3, 18.2 and 10.6 μm in the first independent experiment. We checked the reproducibility by performing the similar experiment for three times (i.e., *n* = 3).

The arithmetic mean of the average values of the distribution, *D*_ave_ = (*d*_ave1_+*d*_ave2_+*d*_ave3_)/3, from three independent experiments were obtained 10.5 ± 0.8 μm for unpurified GUVs, 23.0 ± 1.5 μm for purified GUVs under filter holder 1, and 11.5 ± 1.0 μm for purified GUVs under filter holder 2, where ± indicates standard deviation. The average values of skewness were obtained 1.73 ± 0.25 for unpurified GUVs, 1.21 ± 0.15 for purified GUVs under filter holder 1 and 0.94 ± 0.60 for purified GUVs under filter holder 2. In the similar way, the average values of mode were obtained 7.3 ± 0.8 μm for unpurified GUVs, 18.7 ± 0.5 μm for purified GUVs under filter holder 1 and 9.9 ± 0.8 μm for purified GUVs under filter holder 2. These results suggest that the asymmetry became less for the samples collected from filter holder 2. The less the asymmetry means the probability of finding GUVs of similar size is higher. In addition, the mode of the distribution of GUVs under filter holder 2 is much smaller than in filter holder 1. The average size of GUVs, the skewness and mode of distribution using the combination of 12 μm and 10 μm pores polycarbonate membranes are provided in Table 1.

**Table 1:** The average size of GUVs, skewness and mode of the distribution for different combinations of polycarbonate membrane.

Flow rate (mL/min)	Arrangement of polycarbonate membranes in dual filtration		Average size of GUVs (μm) in filter holder 2	Skewness χ	Mode *X* (μm)
	Filter holder 1	Filter holder 2			
	12 μm	10 μm	11.5 ± 1.0	0.94 ± 0.60	9.9 ± 0.8
1.0	12 μm	8 μm	11.5 ± 0.02	1.64 ± 0.60	8.3 ± 1.5
	10 μm	8 μm	9.0 ± 0.03	0.81 ± 0.01	8.1 ± 0.01

### 3.2. Single filtration with 12 μm pores polycarbonate membrane

**Fig 7:**
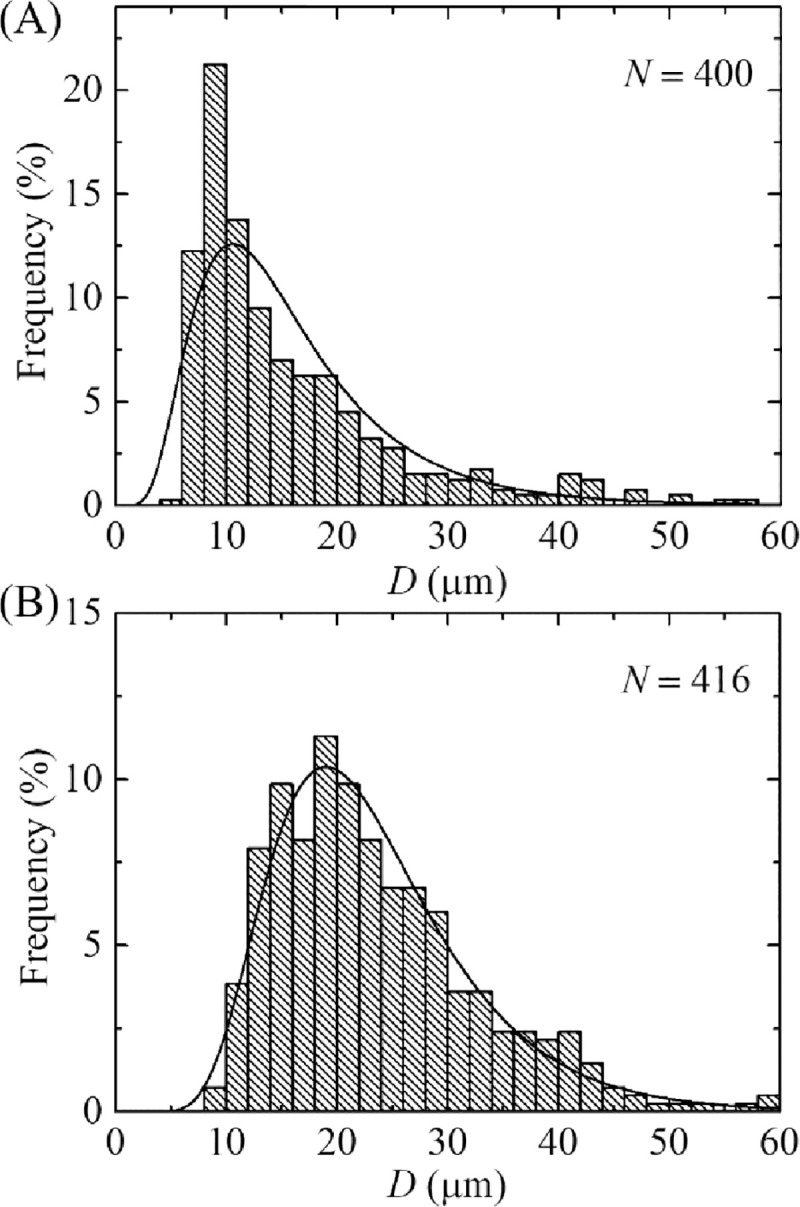
Effects of single filtration on the size distribution of 40%DOPG/60%DOPC-GUVs. The size distribution histogram of the (A) unpurified and (B) purified GUV suspension using a 12 μm pores polycarbonate membrane. The purification was performed at flow rate 1 mL/min for 1 h. The solid line of figures corresponds to fitting with Eq 1.

We also performed the experiment using single filtration technique with 12 μm pores polycarbonate membrane as described in Fig 5. The size distribution histograms of unpurified and purified GUVs are shown in Fig 7A and 7B, respectively. The range of sizes for purified GUVs was 6−59 μm. Both the histograms were fitted using Eq 1, and the average sizes of the distribution were 16.0 μm for unpurified GUVs and 23.5 μm for purified GUVs. The skewness of the distribution of unpurified GUVs was 1.86 and purified GUV suspension was 1.23. We performed 4 independent experiments (i.e., *n* = 4) at same condition and obtained the values of *D*_ave_ as 12.3 ± 2.6 μm for unpurified and 22.8 ± 1.3 μm for purified GUVs. The average values of skewness were obtained 1.65 ± 0.15 for unpurified GUVs and 1.21 ± 0.12 for purified GUVs. The average values of mode were obtained 8.7 ± 1.4 μm for unpurified GUVs and 18.5 ± 0.5 μm for purified GUVs. The values of GUVs size range, average size, skewness and mode were much higher in single filtration compared to that obtained in dual filtration technique, i.e., purified GUVs under filter holder 2. The values of skewness and mode were 1.21 ± 0.12 and 18.5 ± 0.5 μm for single filtration, while the corresponding values were 0.94 ± 0.60 and 9.9 ± 0.8 μm for dual filtration. Moreover, as expected the values obtained in single filtration were very similar to that obtained from filter holder 1 in dual filtration since the pore sizes of the filters were same.

### 3.3 Dual filtration with the combinations of 12 and 8 μm, and 10 and 8 μm pores polycarbonate membranes

**Fig 8:**
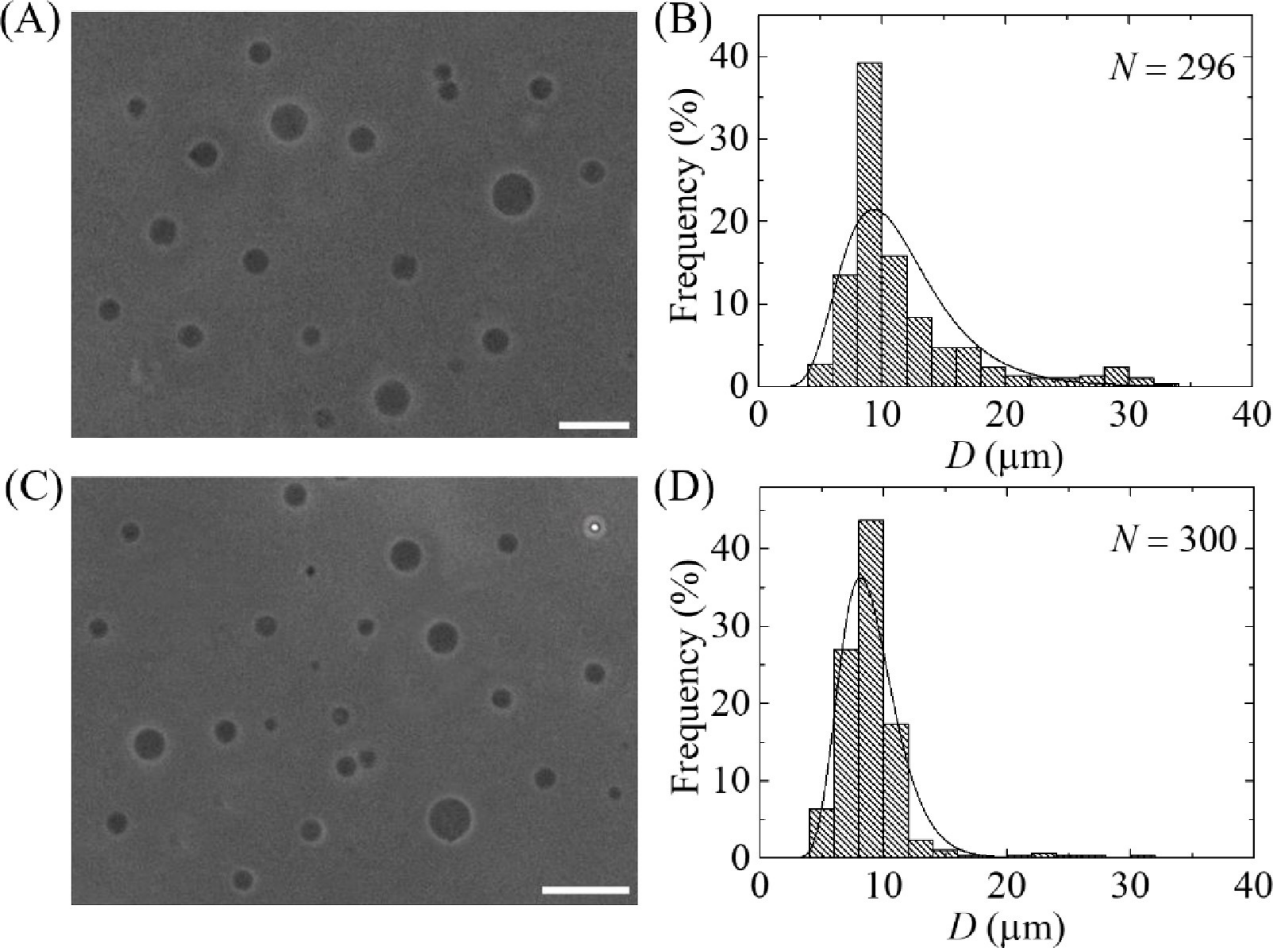
Effects of dual filtration with 12 and 8 μm (case ii), and 10 and 8 μm (case iii) pores polycarbonate membranes on the size and distribution of 40%DOPG/60%DOPC-GUVs. (A) and (B) show the phase contrast image and the corresponding size distribution histogram of GUVs in filter holder 2 for case (ii). (C) and (D) show the phase contrast image and the corresponding size distribution histogram of GUVs in filter holder 2 for case (iii). The purification was performed at flow rate 1 mL/min for 1 h. The bar in the images (A, C) corresponds to a length of 50 μm. The solid line of B and D corresponds to fitting with Eq 1.

So far, we investigated the size distribution of GUVs using case (i), where 12 μm pores polycarbonate membrane used in filter holder 1 and 10 μm pores polycarbonate membrane used in filter holder 2. Here we presented the results of phase contrast images and size distribution of GUVs for case (ii) and case (iii). In case (ii), membrane with 12 μm pores used in filter holder 1 and 8 μm pores used in filter holder 2. In case (iii), membrane with 10 μm pores used in filter holder 2 and 8 μm pores used in filter holder 2. Fig 8A and 8C show the phase contrast images of purified GUVs under filter holder 2 for case (ii) and case (iii), respectively.

The corresponding size distribution histograms were presented in Fig 8B and 8D. The GUVs size distribution were 5.2–32.4 μm for case (ii), whereas it was 4.4–30.3 μm for case (iii). Both the size distributions were well fitted to the Eq 1. From the fitted curves, the average value of the distribution was 11.5 μm and corresponding skewness and mode were 1.23 and 9.3 μm for case (ii). For case (iii), the average value of the distribution was 9.0 μm and corresponding skewness and mode were 0.82 and 8.1 μm. The average sizes of GUVs from *n* = 2 were obtained 11.5 ± 0.02 and 9.0 ± 0.03 μm for case (ii) and (iii), respectively. It is worth to mention that after two independent experiment, the size distribution was significantly similar, thus further experiment was not conducted. The corresponding values of skewness were 1.64 ± 0.6 and 0.81 ± 0.01. Similarly, the corresponding values of mode were 8.3 ± 1.5 and 8.1 ± 0.01 μm. Hence, for all three cases of dual filtration, the distribution followed smaller skewness compared to single filtration. The average size of GUVs, the skewness and mode of distribution using different combinations are provided in Table 1.

### 3.4. Purification of GUVs containing water-soluble fluorescent probe, calcein, using dual filtration technique

**Fig 9:**
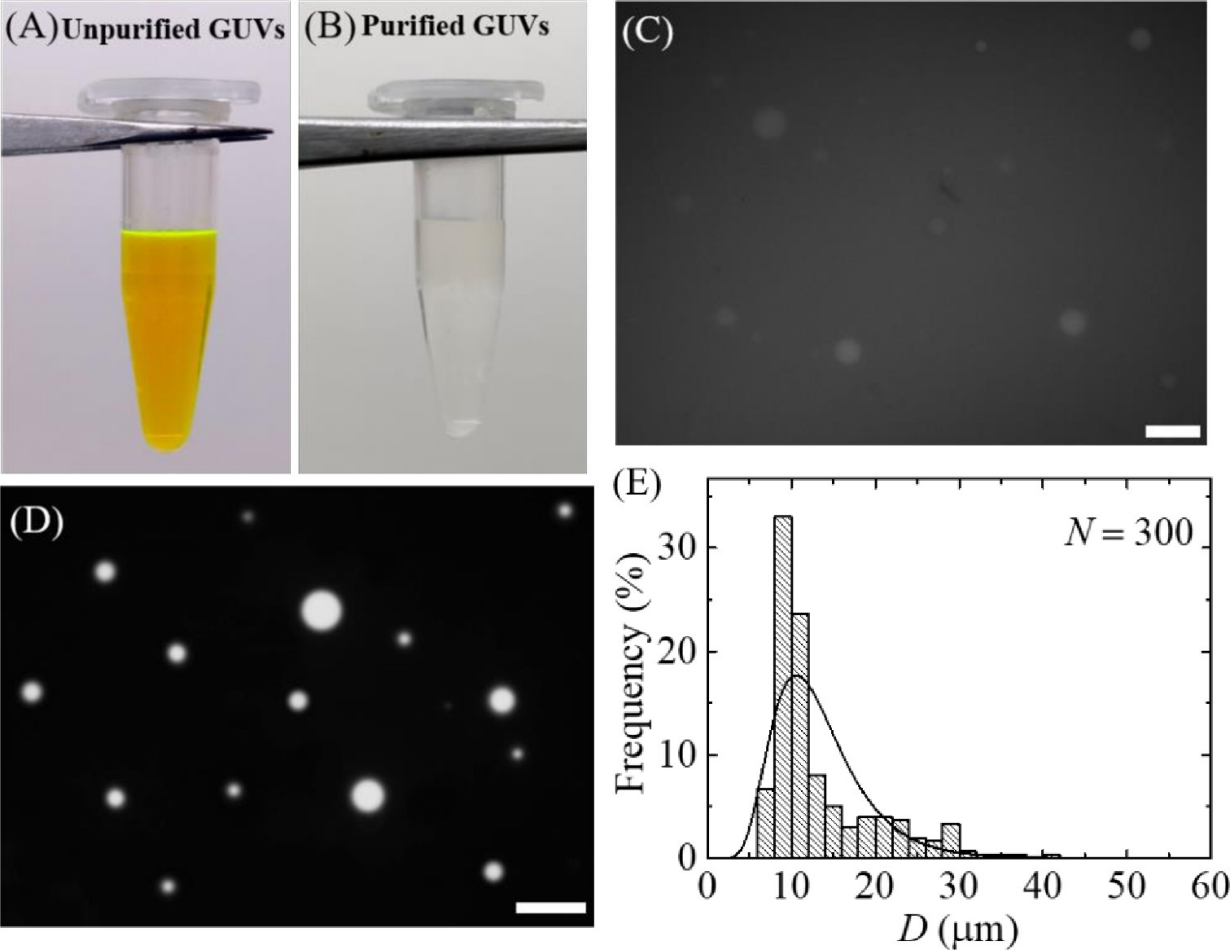
Purification of 40%DOPG/60%DOPC-GUVs containing calcein using the dual filtration technique. (A) GUV suspension before purification (B) GUV suspension after purification. (C) A fluorescence image of unpurified GUV suspension. (D) and (E) show the fluorescence image and the corresponding size distribution histogram GUVs in filter holder 2. The purification was performed at flow rate 1 mL/min for 1 h. The bar in the images (C, D) corresponds to a length of 50 μm. The solid line of E corresponds to fitting with Eq 1.

We investigated the purification of GUVs encapsulating a water-soluble fluorescent probe, calcein (molecular weight 623, Stokes-Einstein radius 0.74 nm) [37]. GUVs were purified using case (i). Before purification, the GUV suspension contained a strong green color due to 1 mM calcein (Fig 9A). After centrifugation, the supernatant of unpurified GUV suspension containing calcein was added into the 60 mL buffer B that was continuously flowing from syringe 1 to filter holder 1 by a peristaltic pump. Hence, the volume ratio of purified GUV suspension and buffer B was 1:22. While running the process of purification, the green color in syringe 1 buffer was diminishing. After purification, the suspension became colourless, indicating a calcein free GUV suspension in buffer B (Fig 9B). Fig 9C shows a fluorescence microscopic image of unpurified GUV suspension. As the calcein solution appeared in GUV suspension, the GUVs were not clearly observed in Fig 9C. The fluorescence microscopic image of purified GUV suspension is shown in Fig 9D, in which the calcein solution is located in the inside of GUVs. The outside became dark, which means that the calcein solution removed from the GUV suspension. The size distribution of purified GUVs (Fig 9E) was fitted to the Eq 1. The average size of distribution was 13.3 μm and the corresponding skewness of distribution was 1.29. The average size of GUVs and the corresponding skewness for *n* = 2 were 13.0 ± 0.5 μm and 1.24 ± 0.60. These values were very similar to that obtained in case (i) in section 3.1. These results suggested that the dual filtration technique can be used for the removal of water-soluble fluorescent probe from the outside of GUV suspension.

The experimental results for removing of water-soluble fluorescence probe was the evidence of removing the smaller vesicles. In our investigations, we could measure the GUVs with diameters greater than 3 μm using optical microscope without any difficulties. Thus, we did not count the vesicles with diameters less than 3 μm. We previously reported that the GUVs with sizes less than 2 μm were not considered as they were too small to visualize [24]. That limitation was also observed by another group [19].

### 3.5. Effects of flow rate on the average size of GUVs in dual filtration technique

**Fig 10:**
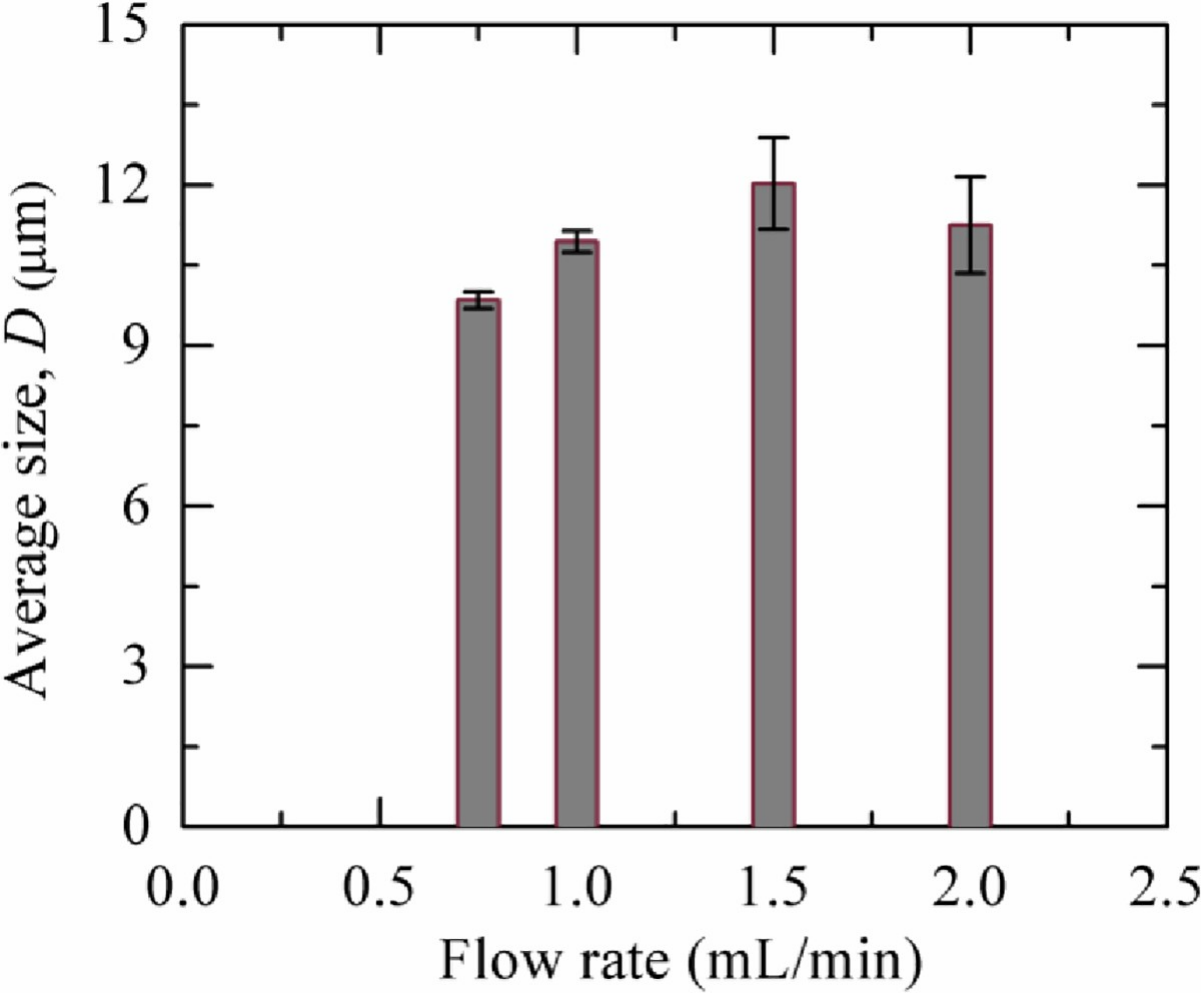
Flow rate dependent average size of purified 40%DOPG/60%DOPC-GUVs using dual filtration technique.

We investigated the dependence of the average GUV size on the flow rate. In Fig 10, it is seen that the average size of GUVs increased at least 10% at flow rate 1.0−2.0 mL/min than 0.75 mL/min. This increment may occur due to the passes of some GUVs with diameters higher than the pores of polycarbonate membrane in filter holder 1 due to higher pressure [6, 38]. As the average size for 1.0–2.0 mL/min became same within the experimental error, those flow rates considered as suitable for dual filtration technique.

### 3.6. Fraction of required sizes GUVs in filter holder 2 compared to the total number of GUVs before filtration

We calculated the fraction of required sizes GUVs under filter holder 2 compared to the total number of GUVs before filtration. It was calculated as, number of GUVs counted within the size of filter pore sizes divided by the total count of GUVs before filtration. As for example, in an experiment of case (i), at first, we measured a total number of 300 GUVs before purification. Then the same number of GUVs was measured under filter holder 2, and counted the GUVs in the range of 10−12 μm. If 150 GUVs were counted, the fraction of required sizes GUVs under filter holder 2 would be (150/300)×100% = 50%. For second and third repetitions of case (i), it were 46% and 50%, respectively. Hence, the average fraction of required sizes GUVs under filter holder 2 was (49 ± 2)×100% for case (i). Similarly, we calculated for case (ii) and case (iii), and presented in Table 2.

**Table 2:** Fraction of required sized GUVs under filter holder 2 compared to the total number of GUVs before filtration for different combination of polycarbonate membranes.

Purification	Flow rate (mL/min)	Arrangement of polycarbonate membranes in dual filtration		Fraction of required sized GUVs under filter holder 2 (%)
		Filter holder 1	Filter holder 2	
Purification once		12 μm	10 μm	49 ± 2
	1.0	12 μm	8 μm	42 ± 2
		10 μm	8 μm	30 ± 1
Purification twice	1.0	12 μm	10 μm	83 ± 1
		12 μm	8 μm	74 ± 3
		10 μm	8 μm	58 ± 2

### 3.7. Effects of repeating dual filtration on the size distribution of GUVs

**Fig 11:**
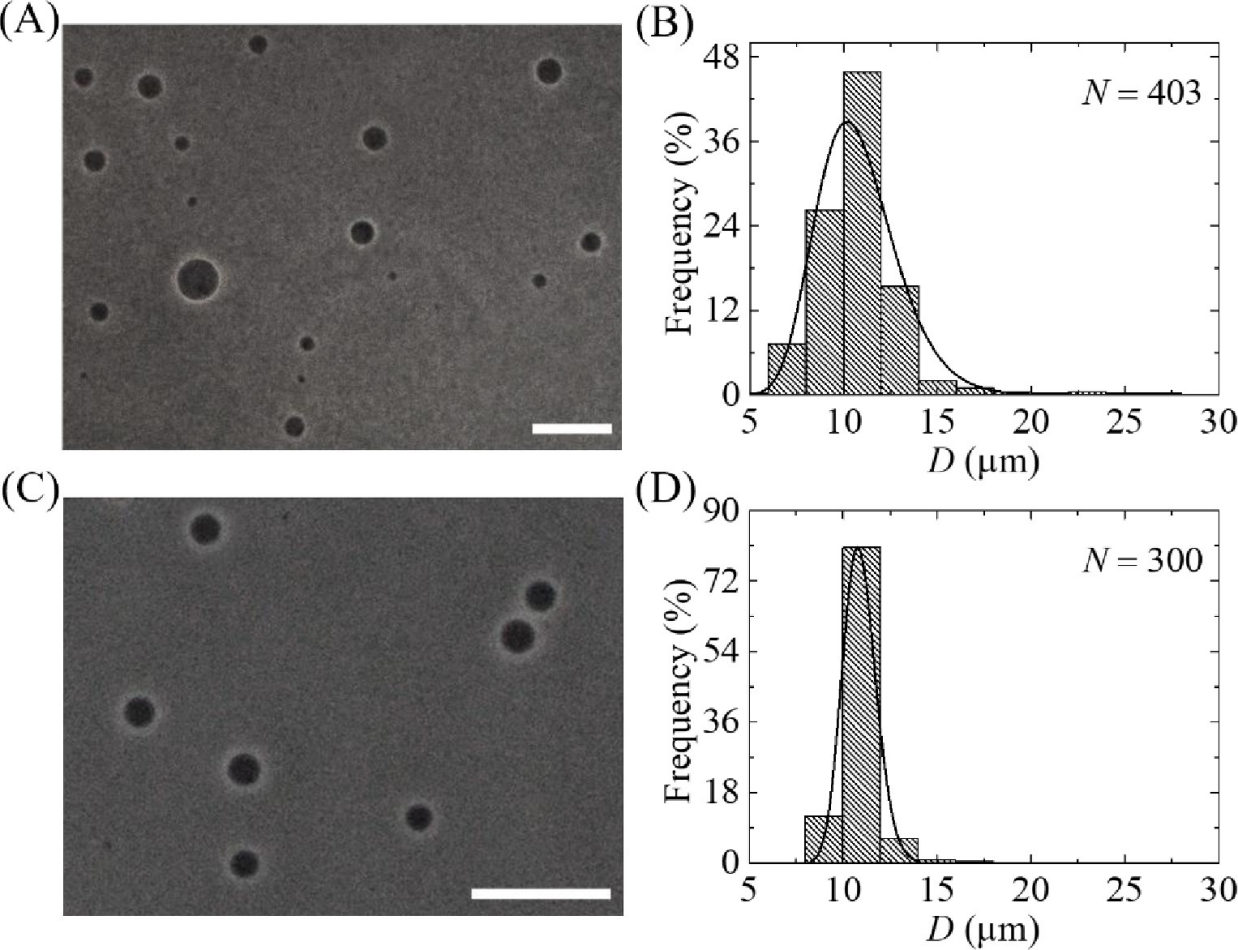
Effects of repeated purification on the phase contrast images and the size distribution of 40%DOPG/60%DOPC-GUVs for case (i). (A) and (B) show the phase contrast image and the corresponding size distribution histogram of GUVs in filter holder 2 after first purification. (C) and (D) show the phase contrast image and the corresponding size distribution histogram of GUVs in filter holder 2 after second purification. The purification was performed at flow rate 1 mL/min for 1 h. The bar in the images (A, C) corresponds to a length of 50 μm. The solid line of B and D corresponds to fitting with Eq 1.

We observed the effects of purification two times on the size distribution of GUVs, i.e., repeat dual filtration of case (i) using the purified GUV suspension as well. The phase contrast images and corresponding size distributions of GUVs after first and second purifications are shown in Fig 11A–11D. In histograms, the ranges of GUVs sizes were 5.9−27.3 μm and 8.1−16.2 μm after first and second purification, respectively. These results indicated that the size distribution of GUVs became narrow after second purification. We fitted the size distributions using Eq 1, and the average values of the size distribution were 10.8 and 12.7 μm for the first and second purification, respectively. The corresponding values of skewness were 0.61 and 0.22. Similarly, the corresponding values of mode were 10.2 μm and 10.7 μm. The average sizes of GUVs were 10.9 ± 0.2 and 13.0 ± 0.5 μm (*n* = 2) while the values of average skewness were 0.58 ± 0.04 and 0.23 ± 0.02 for the first and second purification, respectively. The corresponding values of mode were 10.4 ± 0.3 and 10.8 ± 0.03 μm for the first and second purification, respectively. The value of skewness became lesser after second purification, indicating the higher number of similar sizes GUVs in filter holder 2. In addition, the value of mode in second purification was higher compared to first purification, indicating also the higher number of similar sizes GUVs in filter holder 2. Moreover, the fraction of required sized GUVs under filter holder 2 compared to the total number of GUVs before filtration for case (i) was (83 ± 1)% after second purification, while it was (49 ± 2)% in the first purification as shown in Table 2. The corresponding values for case (ii) and case (iii) are also presented in Table 2. These investigations clearly indicate that if we have needed the more similar size GUVs, it is necessary to perform purification more than once.

### 3.8. Purification of GUVs using an extra filtration with dual filtration

**Fig 12:**
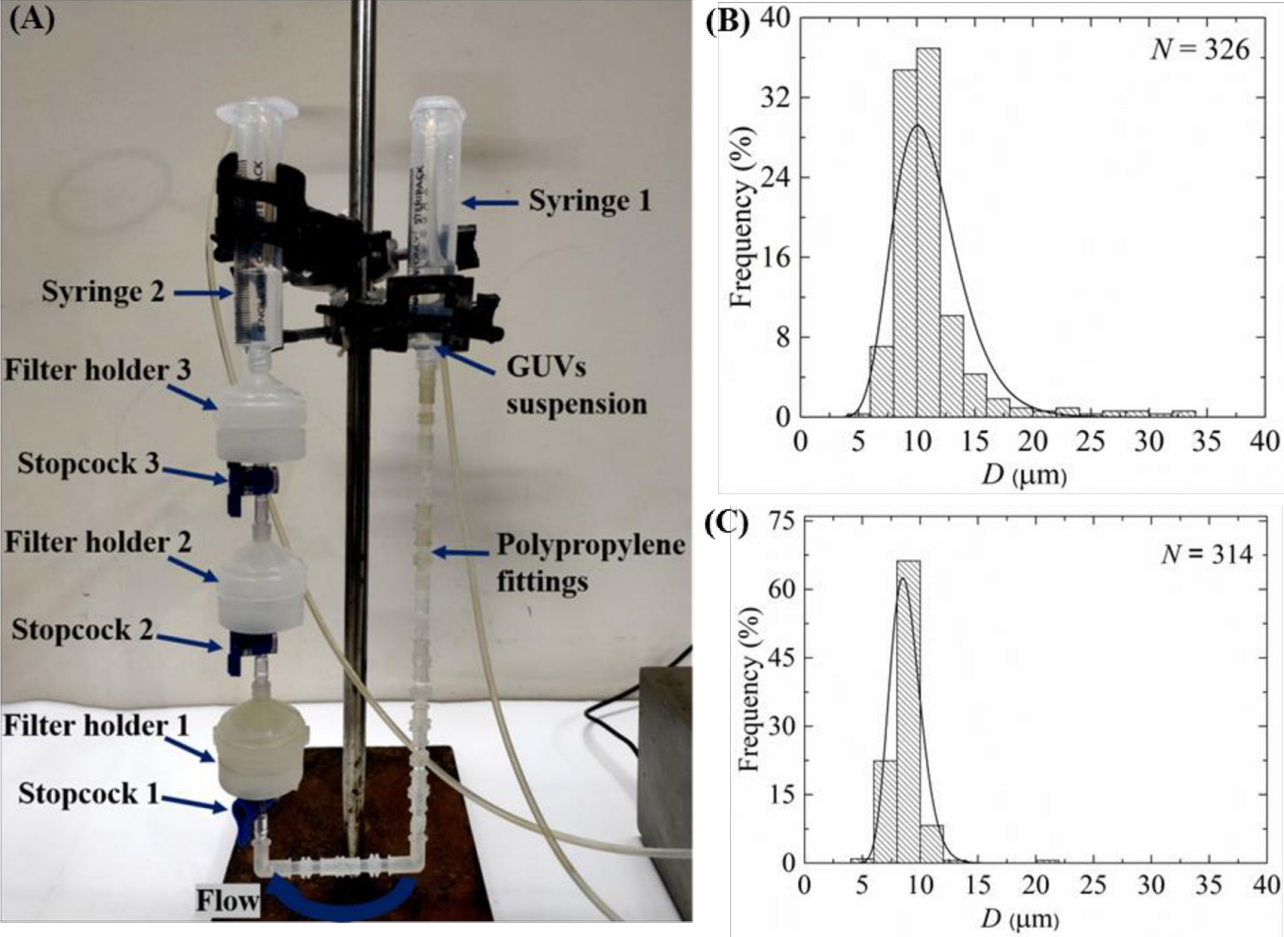
Purification of GUVs using an extra filtration with dual filtration technique. (A) The arrangement of an extra filtration with dual filtration. The size distribution histogram of 40%DOPG/60%DOPC-GUVs in filter holder 2 (B) and filter holder 3 (C). The purification was performed at flow rate 1 mL/min for 1 h. The solid line of B and C corresponds to fitting with Eq 1.

Here we presented the purification of GUVs using an extra filtration with dual filtration as shown in Fig 12A. Here, polycarbonate membranes of 12, 10 and 8 μm pores were used in filter holder 1, 2 and 3, respectively. Fig 12B and 12C show the size distribution histograms of GUVs in filter holder 2 and 3, respectively. These size distributions were very similar to that obtained in dual filtration for case (i) and case (iii) (Figs 6F, 8B and 8D). The average value and the skewness of distribution of GUVs in filter holder 2 were 11.5 μm and 0.59, respectively. The corresponding values were 9.1 μm and 0.84 under filter holder 3. The average size of GUVs and skewness of distribution under filter holder 2 were 11.2 ± 0.4 μm and 0.82 ± 0.06, respectively (*n* = 2), while corresponding values were 8.8 ± 0.2 μm and 0.46 ± 0.07 under filter holder 3.

## 4. Discussion

We developed a GUVs purification technique using dual filtration. The proposed technique purifies the GUVs by removing the smaller vesicles and water-soluble fluorescent probe (i.e., calcein) from the GUV suspension. It gives a narrower size distribution of GUVs compared to single filtration. The size distribution fitted to the lognormal distribution from where the average size of GUVs, skewness and mode of the distribution were calculated. To quantify the change of GUV’s size under various conditions such as unpurified GUVs, GUVs under filter holder 1 and GUVs under filter holder 2, it was necessary to measure the average size of GUVs. We included the skewness measurement to study the asymmetry of the probability distribution of size of vesicles about its mean position. In dual filtration technique, we used three combinations [(case (i), case (ii) and case (iii)] with higher pores of polycarbonate membrane was used in filter holder 1 compared to filter holder 2. Different size distribution of purified GUV suspension was obtained for different combinations of polycarbonate membranes. Interestingly, for all those experiments, we observed presence of some bigger size GUVs than the pores in filter holder 1, and some smaller size GUVs than the pores in filter holder 2 (Figs 6F, 8B and 8D). The reason behind that the membrane of GUVs is elastic and hence, at higher pressure some spherical GUVs alter to prolate and cylindrical shaped vesicles [6, 38], and passed through the pores of polycarbonate membrane in filter holder 1. On the other hand, due to obstruction of smaller vesicles [19, 24] at the pores in filter holder 2, all the GUVs which are smaller than pore sizes would not pass through. The dual filtration technique was also able to purify the GUVs by removing the calcein from the outer suspension of vesicles (Fig 9). Since the Stokes-Einstein radius of calcein is very much smaller (i.e., 0.74 nm) than the size of the pores, therefore, calcein was easily passed through the pores. In dual filtration technique, we obtained a specific size distribution of vesicles rather than a wide range size distribution as obtained in single filtration. The paper [19] used single filtration, but in our case, we proposed dual filtration technique. Therefore, the removing ability of the water-soluble fluorescence probe using the dual filtration technique was necessary.

We investigated the flow rate dependent average size of GUVs and observed that the size of vesicles became slightly higher at flow rate 1.0−2.0 mL/min than 0.75 mL/min (Fig 10). As stated above, GUVs can change their shape at higher pressure [6, 38], and therefore, at flow rate 1.0 mL/min and higher values, some GUVs with diameters greater than the pore sizes were able to pass through.

Interestingly, we observed that the skewness of size distribution for case (ii) was relatively higher than case (i) and case (iii) (Table 1). The higher skewness in case (ii) can be explained in the way that the pore size difference between the filters was higher (i.e., 12–8 = 4 μm), whereas for other cases it was smaller (i.e., 12–10 = 10–8 = 2 μm). As a comparison of dual filtration technique, time duration (i.e., 1 h) was comparable with single membrane filtering [19] and gravity based purification technique [24].

By repeating the dual filtration twice, we got higher fraction of required size GUVs. The reason was that initially the presence of many smaller vesicles hinders the passage of GUVs through the pores, those vesicles were less in the purified GUV suspension. The hindrance became negligible in the second purification as the purified suspension was used. Moreover, a new set of polycarbonate membranes was used in the filter holders before repeating the experiment. The skewness was much smaller (0.23 ± 0.02) in second purification compared to first purification (0.58 ± 0.04) (Fig 11B and 11D). For case (i) such as the combination of 10 μm and 12 μm pores polycarbonate membranes, the fraction of required size GUVs under filter holder 2 compared to the total number of GUVs before filtration was 49% for purification once, while the corresponding value was 83% for purification twice. Therefore, the required sized vesicles contain ~ 49% of the total volume of original population for purification once, while the corresponding value was ~ 83% for purification twice. The remaining amount of lipids was lost. These values varied for other combinations of polycarbonate membrane as presented in Table 2. To get a concentrated similar size of GUVs, second purification is an important choice though it would require double time duration compared to first purification using dual filtration. When we used an extra filtration with dual filtration, we obtained two different size distributions of GUVs (Fig 12B and 12C). One of the main advantages of using these arrangements was that it provided two different size distributions of GUVs at a time in a single purification. However, more experiments are needed to check its performances.

The extrusion-dialysis method [25] was developed for preparing the monodisperse vesicles. This work was similar to our dual filtration technique. In their work, at first, large pore sized (i.e., 5 μm diameter pores) polycarbonate membrane was used for extrusion, and then for dialysis smaller pore sized (i.e., 3 μm diameter pores) polycarbonate membrane was used for removing the smaller vesicles. As a result, they obtained oleate vesicle population between 3–5 μm in diameter. In the same way, POPC vesicles of range of 0.8–1.0 μm was obtained using a different arrangement of polycarbonate membranes. This method integrated two individual methods; one was extrusion (which was generally used for preparing the large unilamellar vesicles-LUVs) and another was dialysis. In the first step of dialysis, it was taken about 1 h (i.e., 5–6 rounds of 5–10 min each) for removing the dye form the vesicle suspension. In the following step, it was taken about 12 h (i.e., at least 6 rounds of minimum 2 h each) for removing the smaller vesicles. The author reported that the overall duration of the process required ~ 24 h. In their work, they prepared multilamellar vesicles (MLVs) by extrusion, and therefore, the resulting vesicles in suspension were also multilamellar.

In our case, we prepared the GUVs using the well-known natural swelling method, and our target was to get the similar sized GUVs having size range higher than that obtained in the extrusion-dialysis method. As for example, in our proposed dual filtration technique, using a combination of 12 μm and 10 μm pores in diameter polycarbonate membranes, it was obtained the GUVs with an average size ~11 μm. In addition, we used 3 different combinations of polycarbonate membranes for understanding the performance of purification. In our case, the time duration for purification was about 1 h, while extrusion-dialysis method required ~ 24 h. Hence, a longer time period is required for getting the purified vesicle suspension in extrusion-dialysis method, which would be hampered the stability of spherical shaped vesicles.

## 5. Conclusion

We developed a dual filtration technique for the purification of GUVs in which a combination of polycarbonate membranes of different size pores was used. This technique would effectively provide a specific and narrow range size distribution of GUVs. It also removed the water-soluble fluorescence probe from the suspension of GUVs. The suitable flow rate for purification was explored. By repeating the filtration, one can obtain even higher number of similar sizes GUVs. This novel purification technique would be promising tool for obtaining a specific size distribution of purified GUVs.
